# Transcriptomic and genomic evidence for *Streptococcus agalactiae* adaptation to the bovine environment

**DOI:** 10.1186/1471-2164-14-920

**Published:** 2013-12-27

**Authors:** Vincent P Richards, Sang Chul Choi, Paulina D Pavinski Bitar, Abhijit A Gurjar, Michael J Stanhope

**Affiliations:** 1 Department of Population Medicine and Diagnostic Sciences, College of Veterinary Medicine, Cornell University, Ithaca, NY 14853, USA; 2 Department of Biological Statistics and Computational Biology, Cornell University, Ithaca, NY 14853, USA; 3 Current address: Institute of Arctic Biology, University of Alaska Fairbanks, Fairbanks, AK 99775, USA; 4 Current address: Merck Animal Health, Global Ruminants Business Unit, Summit, NJ 07901, USA

**Keywords:** *Streptococcus agalactiae*, Bovine adapted, RNAseq, Lactose operon, Lateral gene transfer, Mastitis, Differential gene expression

## Abstract

**Background:**

*Streptococcus agalactiae* is a major cause of bovine mastitis, which is the dominant health disorder affecting milk production within the dairy industry and is responsible for substantial financial losses to the industry worldwide. However, there is considerable evidence for host adaptation (ecotypes) within *S. agalactiae*, with both bovine and human sourced isolates showing a high degree of distinctiveness, suggesting differing ability to cause mastitis. Here, we (i) generate RNAseq data from three *S. agalactiae* isolates (two putative bovine adapted and one human) and (ii) compare publicly available whole genome shotgun sequence data from an additional 202 isolates, obtained from six host species, to elucidate possible genetic factors/adaptations likely important for *S. agalactiae* growth and survival in the bovine mammary gland.

**Results:**

Tests for differential expression showed distinct expression profiles for the three isolates when grown in bovine milk. A key finding for the two putatively bovine adapted isolates was the up regulation of a lactose metabolism operon (Lac.2) that was strongly correlated with the bovine environment (all 36 bovine sourced isolates on GenBank possessed the operon, in contrast to only 8/151 human sourced isolates). Multi locus sequence typing of all genome sequences and phylogenetic analysis using conserved operon genes from 44 *S. agalactiae* isolates and 16 additional *Streptococcus* species provided strong evidence for acquisition of the operon via multiple lateral gene transfer events, with all *Streptococcus* species known to be major causes of mastitis, identified as possible donors. Furthermore, lactose fermentation tests were only positive for isolates possessing Lac.2. Combined, these findings suggest that lactose metabolism is likely an important adaptation to the bovine environment. Additional up regulation in the bovine adapted isolates included genes involved in copper homeostasis, metabolism of purine, pyrimidine, glycerol and glucose, and possibly aminoglycoside antibiotic resistance.

**Conclusion:**

We detected several genetic factors likely important in *S. agalactiae*’s adaptation to the bovine environment, in particular lactose metabolism. Of concern is the up regulation of a putative antibiotic resistance gene (GCN5-related N-acetyltransferase) that might reflect an adaptation to the use of aminoglycoside antibiotics within this environment.

## Background

In addition to causing severe invasive infections in adults and neonates (e.g. pneumonia, meningitis, and septicemia) [1-3], *Streptococcus agalactiae* (Group B *Streptococcus*; GBS) is a major cause of bovine mastitis [4,5], which is the dominant health disorder affecting milk production in the dairy industry, and is responsible for significant financial losses worldwide [6-11]. *S. agalactiae* has been isolated from a diversity of vertebrate hosts ranging from humans to crocodiles and fish [12-14], and there is considerable evidence for host adaptation among strains, with both human and bovine sourced isolates showing a high degree of genetic distinctiveness [15-20]. Therefore, the objectives of this study were to (i) assess how the genomic gene expression of bovine sourced *S. agalactiae* (putative bovine adapted) responded to growth in bovine milk when compared to human sourced *S. agalactiae*, and (ii) place our findings into a wider context via comparison of over 200 additional *S. agalactiae* genome sequences obtained from a variety of host species.

A principle now generally regarded as common to most or all bacteria is that of the pan-genome, which is comprised of both a set of core and dispensable genes, with only the former present in all isolates of that species [21]. There is now abundant evidence to support the view that the dispensable genes are fundamental to adaptive and phenotypic differences between strains [20,22-28]. Earlier genomic studies of *S. agalactiae* gene expression used microarrays and did not focus on potential ecotypic adaptation. [29-33]. Here we present the first comparative transcriptomic study of *S. agalactiae* based on RNAseq data. The comparison involves three *S. agalactiae* strains representing distinct ecotypes to elucidate possible genetic factors/adaptations likely important for *S. agalactiae* growth and survival in the bovine mammary gland. Our findings reveal several genetic factors likely important in *S. agalactiae*’s adaptation to the bovine environment, in particular, genes involved in carbohydrate metabolism.

## Methods

### Strain selection

The putative bovine adapted *S. agalactiae* strain used in this study (FSL S3-586) was obtained from a quarter milk sample isolated from a cow with mastitis in Wayne County, NY, USA [18]. The strain was isolated in 2001 and is MLST sequence type (ST) 67. ST-67 has been frequently isolated from cows suffering with mastitis [15]. The human sourced strain (CCUG 37738) was isolated from the blood of a female newborn with sepsis in Göteborg, Sweden (1994). The strain was determined here to be ST-19 using its genome sequence (GenBank accession number: ALQP01000000). ST-19 has been frequently isolated from human sources [15,34-36]. *S. agalactiae* strains from the ST-23 lineage have possibly the widest host range. Strains have been isolated from humans, cattle, dogs, crocodiles, and grey seals, with the ST showing high frequency in both human and bovine environments [5,12,13,15,34-36]. Given that strains from this lineage might be adapted to both human and bovine environments, we also included a strain with this ST in our analysis. We selected strain NEM316, as this strain has been included in numerous previous studies (including whole-genome transcriptome analysis) and its genome has been sequenced [12,15,29,32,35,37-39]. While the strain has been described in the literature as being a human isolate, Sørensen et al. [35] showed its isolation source to be unknown.

No experimental research on vertebrates or any regulated invertebrates was performed. Compliance with the ARRIVE (Animal Research: Reporting In Vivo Experiments) guidelines is not applicable.

### Bacteria culturing

All strains were grown, in triplicate, in untreated bovine milk and Todd Hewitt Broth with 0.5% yeast extract (THY) at 37ºC and normal atmospheric conditions. Bovine milk with low somatic cell count (< 200,000 cells/mL) was collected and pooled from four cows (seven quarters total), approximately 30 minutes prior to inoculation. Overnight cultures of the strains were used to inoculate the milk and THY media to 1:100 dilution (500 μL overnight culture in 50 mL media). Cultures were grown to mid-exponential phase and harvested.

Growth curve measurements for each strain were obtained using the drop plate method [40]. For each one-hour time point, serial dilutions were made (in PBS pH 7.4) and plated (five drops of 10 μL in duplicate for each of four to six dilutions) on TSAII with 0.5% sheep blood and incubated overnight. After 24 hours, colonies were counted and CFU/μL was calculated. Drops were considered countable if they contained 3-30 colonies. Standard error for each pair of replicates at each time point was calculated (for some time points, only one drop was countable, precluding standard error calculation).

### Lactose fermentation

Strains were grown at 37°C in 7 mL Phenol Red Broth Base with Meat Extract (HiMedia) supplemented with 1% lactose (BD Difco). After 7 days the color of the media was noted. The media changed color to either yellow or orange. Two pH measurements were taken for each. For yellow the average pH was 4.6, for orange the average pH was 6.5. A color change to yellow was taken as an indication of lactose fermentation.

### RNA extraction, cDNA library construction, and sequencing

Milk cultures at mid-exponential phase were centrifuged at 3,500 rpm and 4ºC for 30 minutes to pellet the bacteria. Whey and fat were removed and the pellet re-suspended in 9.0 mL of 1:2 mix of phosphate buffered saline (PBS) and RNAprotect (Qiagen). The solution was filtered through a five-micron filter to remove intact bovine cells while leaving behind *S. agalactiae* cells (size ≤ 1 micron). Cells were centrifuged at 5000 rpm for 10 minutes, supernatant removed, and the pellet frozen at -80ºC. THY cultures at mid-exponential phase were centrifuged at 3,500 rpm and 4ºC for 30 minutes to pellet the bacteria. The pellet was re-suspended in 1.0 mL RNAprotect. Following a second centrifugation at 5,000 rpm for 10 minutes, the supernatant was removed and the pellet frozen at -80ºC. All pellets were re-suspended in 280 μL of TE and combined with 300 μL of acidic phenol:choloroform (Ambion) and 250 μL of 0.1 mm glass beads (BioSpec Products). Cells were mechanically disrupted and centrifuged at 13,200 rpm for 10 minutes to separate them. 200 μL of the supernatant was combined with 700 μL Qiagen RLT buffer containing 7 μL β-mercaptoethanol, and transferred to a Qiagen RNeasy Mini kit column. The extraction was completed following the Qiagen protocol, which included an on-the-column DNAse digest (Qiagen). Trace mammalian RNA was removed from extracted RNA samples using the MICROBEnrich™ Kit (Ambion). Samples were depleted of rRNA using the MICROBExpress™ Bacterial mRNA Enrichment Kit. cDNA libraries were constructed using the TruSeq RNA Sample Prep Kit (Illumina) and sequenced using Illumina Hiseq 2000 (100 bp reads, single end) (six samples/lane). Two separate replicates (one each for strains FSL S3-586 and NEM316) grown in broth were excluded from sequencing due to suspected cross contamination. Summary sequencing statistics are shown in Table 1.

**Table 1:** Summary Illumina sequencing statistics

**Strain**	**Growth environment**	**Replicate**	**Total number of reads**	**Number of aligned reads**
NEM316	Milk	1	29,089,124	27,350,545
NEM316	Milk	2	40,554,665	38,161,713
NEM316	Milk	3	35,497,113	33,357,550
NEM316	Broth	1	23,013,312	21,250,407
NEM316	Broth	2	23,145,072	21,484,496
CCUG 37738	Milk	1	20,106,316	7,516,242
CCUG 37738	Milk	2	16,800,749	6,494,892
CCUG 37738	Milk	3	28,308,724	11,028,696
CCUG 37738	Broth	1	44,274,054	19,357,826
CCUG 37738	Broth	2	27,893,761	11,196,100
CCUG 37738	Broth	3	17,763,818	7,925,653
FSL S3-586	Milk	1	29,138,705	8,033,022
FSL S3-586	Milk	2	27,373,266	4,909,064
FSL S3-586	Milk	3	16,688,288	6,786,044
FSL S3-586	Broth	1	15,172,203	4,505,824
FSL S3-586	Broth	2	21,093,103	6,750,884

The average proportion of reads passing the Illumina quality filter for each replicate was 94.6% (range = 93-97%). The number of aligned reads excludes reads ambiguously aligned to multiple locations within the respective genome sequence.

### Differential gene expression, functional annotation, and clustering

Adaptor sequences were removed from Illumina reads using cutadapt [41]. Reads for each strain were then mapped to respective genome sequences using BWA [42]. Genome sequences were obtained from GenBank (FSL S3-586: ANCM01000000, CCUG 37738: ALQP01000000, NEM316: NC_004368). Short read alignments were converted to readable formats using SAMtools [43], and the number of reads aligned to annotated genes was counted using R [44]. Significant differences in expression between growth in milk and broth were determined using DEseq [45]. The false discovery rate (FDR) procedure of Benjamini and Hochberg [46] was used to correct for multiple hypothesis testing (*FDR* = 0.05). Gene Ontology (GO) terms were assigned to genes showing significant differential expression using Blast2GO v.2.5.0 [47]. These genes were also assigned GO-Slim terms using the generic GO Slim (http://www.geneontology.org/GO_slims/goslim_generic.obo). GO Slim is a reduced version of the full GO that contains a sub-set of more general GO terms and excludes the more fine-grained specific terms. This approach provides a broad overview of the ontology and gene product function for genomic data.

Genes for all three strains were delineated into homologous clusters using the MCL algorithm [48] as implemented in the MCLBLASTLINE pipeline (available at http://micans.org/mcl). The pipeline uses Markov clustering (MCL) to assign genes to homologous clusters based on a BLASTp search between all pairs of protein sequences using an *E* value cut-off of 1e-5. The MCL algorithm was implemented using an inflation parameter of 1.8. Simulations have shown this value to be generally robust to false positives and negatives [49].

### Phylogenetic analysis

The *lacGEFDCBA* genes from the Lac.2 operon (see Results and Discussion) for 60 *Streptococcus* strains (17 species) (Additional file 1) were aligned using MAFFT v6.814b [50] as implemented in Geneious v5.5.3 [51]. A Maximum Likelihood phylogeny with 500 bootstrap replicates was constructed using PhyML v3.0 [52] and the GTR + I + G substitution model, which was determined as the best fit for the data using Modeltest v3.7 [53].

## Results and discussion

### Differential gene expression

The human isolate showed considerably more differential expression when grown in bovine milk than the other two strains. Specifically, 305, 34, and 48 genes for the human isolate, bovine isolate, and NEM316 showed significantly more expression in bovine milk when compared to broth (up regulation) (see Additional file 2). This represents 14.4%, 1.4%, and 2.3% of the total number of genes in each genome respectively. The isolates had similar numbers of genes with less expression in milk relative to broth (down regulated): human = 298 (14.0%), bovine = 43 (1.8%), and NEM316 = 25 (1.2%).

**Figure 1:**
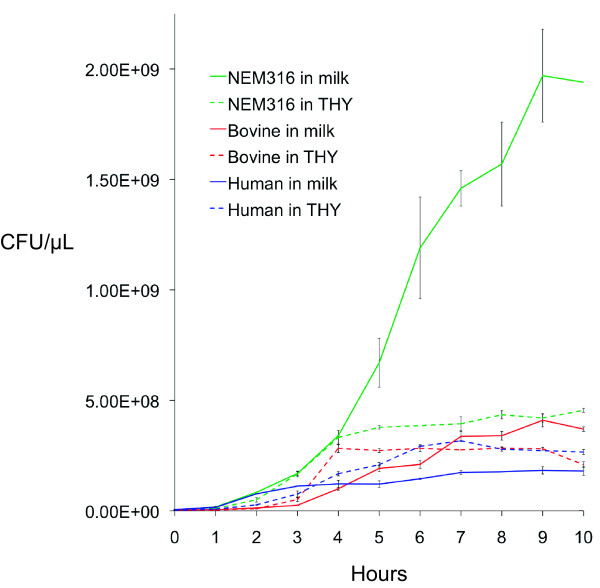
Growth curves, with standard error bars, for strains FSL S3-586 (bovine sourced isolate), CCUG 37738 (human sourced isolate), and NEM316 grown in bovine milk (solid line) and THY broth (dashed line).

The number of genes up regulated was not correlated with growth. For example, in milk, final CFUs/μL were highest for NEM316, followed by the bovine isolate, and lastly the human isolate, with NEM316 showing considerably more growth than the other two isolates (Figure 1). In broth, the isolates showed less variation in growth. Nevertheless, NEM316 again showed the highest growth, with the other two strains showing approximately equal growth. Both NEM316 and the bovine isolate showed higher growth in milk compared to broth. However, the difference was far more pronounced for NEM316. Conversely, the human isolate showed more growth in broth than milk. Combined, these observations suggest that NEM316 and the bovine isolate are better adapted to the bovine environment than the human isolate.

**Figure 2:**
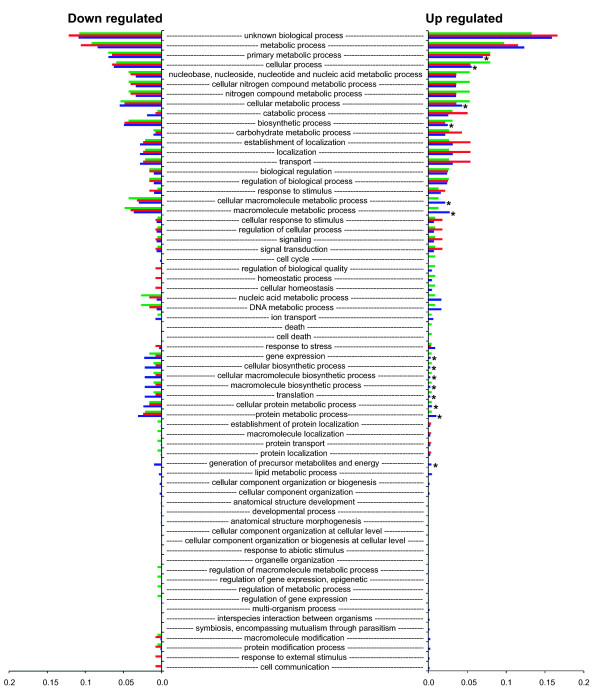
**Proportion of each of the GO Slim biological process terms assigned to genes showing differential expression.** Green = FSL S3-586 (bovine sourced isolate), red = NEM316, blue = CCUG 37738 (human sourced isolate). Right side of the chart shows proportions for terms assigned to genes that showed significantly more expression in milk compared to broth (up regulated). Left side of the chart shows proportions for terms assigned to genes that showed significantly more expression in broth compared to milk (down regulated in milk). An asterisk shows terms for the human isolate that were significantly under represented for up regulated genes (FDR correction of 0.05).

**Figure 3:**
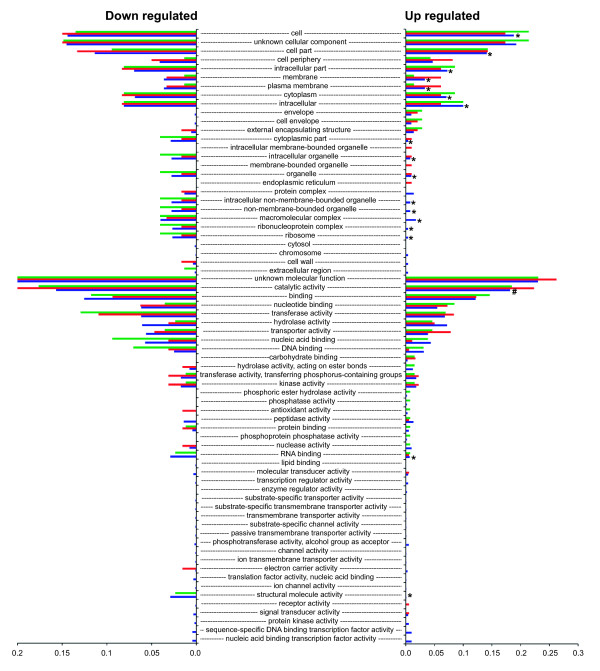
**Proportion of each of the GO Slim molecular function and cellular component terms assigned to genes showing differential expression.** From the top of the chart, the first 28 terms are cellular component. Green = FSL S3-586 (bovine sourced isolate), red = NEM316, blue = CCUG 37738 (human sourced isolate). Right side of the chart shows proportions for terms assigned to genes that showed significantly more expression in milk compared to broth (up regulated). Left side of the chart shows proportions for terms assigned to genes that showed significantly more expression in broth compared to milk (down regulated in milk). An asterisk shows terms for the human isolate that were significantly underrepresented for up regulated genes when compared to down regulated genes. The # symbol shows a single term for the human isolate that was significantly over represented for up regulated genes (FDR correction of 0.05). Note: the x-axis scale for down regulated genes ends at 0.2, whereas the scale for the up regulated genes ends at 0.3.

Figures 2 and 3 show the proportion of each of the GO Slim terms that were assigned to the genes showing differential expression. Proportions are relative to the total number of terms assigned (genes can be assigned multiple GO terms). Therefore, the chart shows the proportional distribution of functional categories for the genes differentially expressed for each isolate. The right side of the chart shows proportions for terms assigned to genes that were up regulated, whereas the left side shows proportions for genes that were down regulated. For each term, Fisher exact tests comparing the number of up regulated genes to the number of down regulated genes detected no significant differences for the bovine isolate or NEM316 (FDR correction of 0.05). However, tests for the human isolate detected 33 terms that were significantly underrepresented for up regulated genes and one term that was over represented (Figures 2 and 3). Of these terms, 16 were for biological processes, 15 were for cellular component, and three were for molecular function. For the biological processes terms, metabolic and biosynthesis processes were the most frequent. For example, there were eight terms associated with metabolic processes and five terms associated with biosynthesis processes. The remaining three terms were for translation, gene expression, and generation of precursor metabolites and energy. These findings suggest a significant reduction in metabolic activity for the human isolate when grown in bovine milk.

The human isolate was also distinctive regarding genes involved in response to stress. Specifically, the following four genes were up regulated: a CSD family cold shock protein (SAG0061_02621), a universal stress family protein (SAG0061_03696), a heat shock protein *GrpE* (SAG0061_10653), and a heat-inducible transcription repressor (SAG0061_10658). The isolate showed a similar stress response to growth in broth, with the following three stress response genes showing up regulation: a universal stress family protein (SAG0061_03421), a heat shock protein *HtpX* (SAG0061_03931), and a molecular chaperone *DnaK* SAG0061_10648. In contrast, the bovine isolate showed no up regulation for stress response genes in either environment and NEM316 showed up regulation for just one gene when grown in milk: a cold shock protein (gbs2053). Concordant with the growth patterns in milk, these results also suggest that the bovine isolate and NEM316 are better adapted to the bovine environment than the human isolate. However, while these results also suggest that the bovine isolate and NEM316 are better adapted to the broth environment than the human strain, this was not entirely reflected by the growth patterns in broth as the bovine and human isolates showed similar growth in this environment. In other words, despite the human isolate’s strong stress response in broth, it was still able to grow as well as the bovine isolate in this environment.

Interestingly, strain NEM316 does not appear as well-adapted to human blood as it does to milk. For example, Mereghetti et al. [29] reported that after 90 minutes growth at 37°C and 40°C the following stress response genes were up regulated: a universal stress family protein (gbs1721), two general stress proteins (gbs1202 and gbs1204), a chaperone (gbs0625), a ClpL protease (gbs1376gbs), and a stress response regulator (gbs0756). Conversely, NEM316 might be as equally well adapted to human amniotic fluid, as it appeared to be to milk, as Sitkiewicz et al. [32] reported that it showed down regulation for all stress response genes when grown in this environment (with the exception of one gene [gbs2029–chaperonin GroEL] that showed moderate up regulation).

### Lactose operon

**Figure 4:**
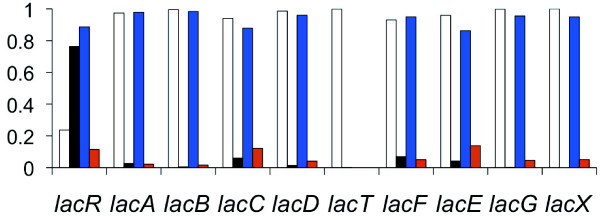
**Bar chart showing the relative proportion of the abundance of reads aligned to each Lac.2 gene for strains NEM316 and FSL S3-586 when grown in milk and then broth.** Read abundance is the mean of the normalized count of reads for each set of replicates. Chart shows abundance as follows: white bar = proportion of reads aligned for NEM316 when grown in milk, black bar = proportion of reads aligned for NEM316 when grown in broth, blue bar = proportion of reads aligned for FSL S3-586 when grown in milk, red bar = proportion of reads aligned for FSL S3-586 when grown in broth. *LacT* was absent in strain FSL S3-586 (see Results and Discussion).

Both the bovine isolate and NEM316 showed up regulation for a shared eight or nine gene operon that has been shown to be involved in the transport and metabolism of lactose [54] (Figure 4). The operon corresponds to what is referred to as Lac.2 [55] and utilizes the phosphoenolpyruvate (PEP)-dependant sugar-phosphotransferase system (PTS). With one exception, the clustering analysis showed all genes in the operon for each of the two isolates to be homologous. The exception was NEM316, which possessed an additional gene: *lacT*. Another difference between the two operons was that *lacR* for the bovine isolate was orientated in the opposite direction to the remainder of the operon. Gene order for the operon was as follows: *lacRABCDFEGX* (bovine isolate), *lacRABCDTFEGX* (NEM316). Both *lacR* and *lacT* may have regulatory functions. For example, *lacR* is a putative negative regulator of the operon that likely functions by binding to the promoter in the absence of lactose [54,56,57], and in contrast to the remainder of the operon, this gene was not up regulated. *lacT* is an antiterminator, which may function to regulate downstream expression of *lacFEGX*. For *Lactobacillis casei,* the *lacT* gene product is believed to bind to a ribonucleic antiterminator (RAT) motif within an mRNA secondary structure that prevents the formation of a rho-independent terminator stem-loop structure that would otherwise terminate downstream transcription [58]. For NEM316, *lacT* was up regulated and a search of the upstream 392 bp intergenic region using the ARNold webserver (http://rna.igmors.u-psud.fr/toolbox) detected a putative 65 bp rho-independent terminator motif with a stem-loop free energy of -8.26 kcal/mol. In addition, a putative -10 promoter consensus sequence (TATAAT) was detected starting 98 bp upstream of the start of the terminator. Although we could not detect a motif resembling the proposed RAT consensus sequence of Brown and Thompson [59], previously detected in *Streptococcus mutans*[60], we did detect a 35 bp imperfect inverted repeat that was 100 bp upstream of the rho-independent terminator that might function as a RAT.

While both the putatively bovine adapted isolates possessed Lac.2, the human isolate lacked it, and previous examination of genome sequences for seven human sourced isolates and one bovine sourced isolate also showed that Lac.2 was only present in the bovine sourced isolate [20]. Here we used BLASTn (*E* value cut-off of 1e-5) to survey an additional 202 *S. agalactiae* genome sequences available on GenBank (see Additional file 1) for the presence of Lac.2. These strains were isolated from a range of vertebrate hosts: human = 151, bovine = 35, tilapia = 13, dolphin = 1, bullfrog = 1, and grey seal = 1. Lac.2 was present in 42 isolates. This included all 35 bovine sourced isolates and seven human sourced isolates. Again using BLASTn, we determined the MLST ST for each isolate (including the bovine and human isolates analyzed using RNAseq data). All of these genome sequences were WGS, and as a consequence, MLST alleles were occasionally truncated. Therefore, we were unable to determine allele profiles for 21 isolates. Of the seven human sourced isolates that possessed Lac.2, three were ST-88, two were undetermined, and the remaining two were ST-25 and ST-103. ST-88 and ST-25 belong to clonal complex 23, which also includes ST-23 (NEM316). These results clearly show a very strong correlation between Lac.2 and the bovine environment (all bovine sourced isolates possessed the operon).

We identified 17 different STs for the bovine isolates (Additional file 1) and previous studies have shown these STs to not cluster together in phylogentic analyses [35,61]. Furthermore, there were numerous examples where the possession of Lac.2 was not conserved for all isolates of the same ST. For example, ST-1 occurred 19 times, yet only three of the isolates with this ST possessed Lac.2 (Additional file 1). Other examples were ST-23 (n = 16, Lac.2 = 1), ST-7 (n = 9, Lac.2 = 1), ST-88 (n = 6, Lac.2 = 3), and ST-2 (n = 5, Lac.2 = 1). Combined, these findings strongly suggest that acquisition of Lac.2 was via lateral gene transfer (LGT) rather than vertical inheritance. LGT of this operon is further supported by previous work showing that Lac.2 was likely exchanged between a bovine sourced isolate of *S. agalactiae* and another mastitis causing pathogen *Streptococcus dysgalactiae* subsp. *dysgalactiae* via an integrative conjugative element (ICE) [20]. Furthermore, Lac.2 is also within an ICE for NEM316 and another species of *Streptococcus* (*Streptococcus canis* - FSL S3-227) [62]. Strain FSL S3-227 was isolated from a cow with an intra-mammary infection that belonged to a dairy herd experiencing an outbreak of *S. canis* induced mastitis [63].

### Evolution of Lac.2

**Figure 5:**
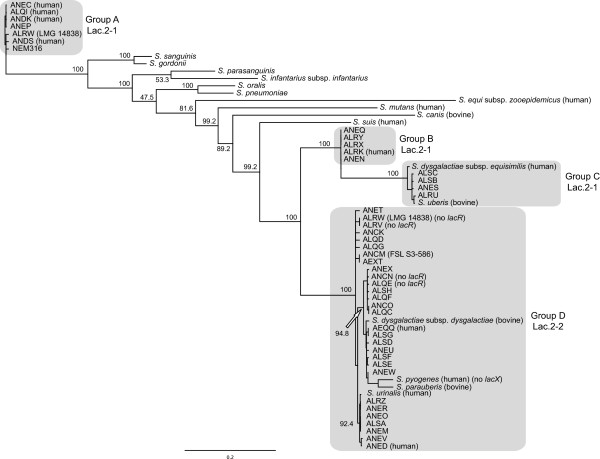
**Maximum likelihood phylogeny showing relationship among *lacGEFDCBA *genes from the Lac.2 operon for 44** ***S. agalactiae *strains and 16 additional *Streptococcus *species.***S. agalactiae* strains are labeled using their 4-letter accession prefix. See Additional file 1 for cross reference to all strain names. Four major *S. agalactiae* groups are shaded.

We further explored the evolutionary history of Lac.2 by aligning the operon from each *S. agalactiae* isolate to 16 additional *Streptococcus* species representing a range of phylogenetic groups (mitis, sanguinis, mutans, pyogenic, and bovis) and then constructed a maximum likelihood phylogeny (see Methods, Figure 5, and Additional file 1). The genes *lacX*, *lacT*, and *lacR* were not consistently present in all isolates nor species and were therefore excluded from the analysis. Within the phylogeny, the Lac.2 sequences for *S. agalactiae* formed four major groupings (A, B, C, and D) that were strongly supported. Three (B, C, and D) clustered together, while the fourth (A), which contained NEM316, was very distantly related (10 of the additional *Streptococcus* species separated them). As described above, *S. agalactiae* isolates possessed one of two different types of Lac.2 operon. Here we designate them Lac.2-1 (*lacT* present and *lacR* orientated in the same direction as the remainder of the operon) and Lac.2-2 (*lacT* missing and *lacR* orientated in the opposite direction). Four isolates within group D lacked *lacR* (Additional file 1 and Figure 5). When the two types of Lac.2 were overlain on the phylogeny, they clustered separately, with groups A, B, and C exclusively containing Lac.2-1 and group D exclusively containing Lac.2-2, suggesting distinct evolutionary histories for the Lac.2 types. Four of the additional *Streptococcus* species fell within group D (*S. dysgalactiae* subsp. *dysgalactiae*, *Streptococcus pyogenes*, *Streptococcus parauberis*, and *Streptococcus urinalis*) and two within group C (*Streptococcus dysgalactiae* subsp. *equisimilis* and *Streptococcus uberis*). The close relationship between these additional species and the *S. agalactiae* group they fell within, compared to the much larger evolutionary distance among the *S. agalactiae* groups lends further support for the lateral exchange of Lac.2 between *S. agalactiae* and other *Streptococcus* species. It’s noteworthy that *S. dysgalactiae* subsp. *dysgalactiae*, *S. uberis*, and *S. parauberis* are major causes of mastitis and were all isolated from the bovine environment, suggesting this exchange may have occurred within this environment. However, three additional *Streptococcus* species, all isolated from the human environment, also fell within groups C (*S. dysgalactiae* subsp. *equisimilis*) and D (*S. pyogenes* and *S. urinalis*), raising the possibility that exchange may have also occurred in the human environment. One *S. agalactiae* strain (LMG 14838) was particularly interesting, as it possessed both types of Lac.2, with the phylogeny showing them to be highly divergent, likely reflecting acquisition of the operon on separate occasions from different species. None of the additional species included in the phylogeny fell within groups A and B. Therefore, in an attempt to identify possible donor species, we performed a BLASTn search of the nr database at NCBI using a representative sequence from each group. The best hit for each group was *Streptococcus sanguinis* (group A) and *S. dysgalactiae* subsp. *equisimilis* (group B). Unfortunately, these two species were already included in our phylogeny and were the closest species to each group. Consequently, the likely donor species for these two groups remains unknown.

### The role of Lac.1

The previous examination of genome sequences for seven human and one bovine sourced isolate showed that in addition to Lac.2, all *S. agalactiae* isolates possessed a second lactose operon Lac.1: *lacR*-IIA-IIB-IIC-neuraminidase-*ABCDX*[20]. The clustering analysis performed here showed that although genes *ABCDX* were homologous with those in Lac.2, the operon lacked *lacG*, a cytoplasmic phospho-β-galactosidase that hydrolyzes the lactose-6-phosphate produced when lactose is transported into the cell via the PTS. The lactose-6-phosphate is hydrolyzed to galactose-6-phosphate prior to it passing through the tagatose-6-phosphate pathway. Consequently, the absence of *lacG* in Lac.1 suggests that the operon’s primary function is not the metabolism of lactose, and Loughman and Caparon [56] suggested a virulent role for the operon in *S. pyogenes.* Similarly, Lac.1 in *S. agalactiae* contains neuraminidase, which has been implicated in virulence, also suggesting an alternative role for the operon [64,65]. However, a BLASTn search for *lacG* in the 202 genome sequences showed it to be present somewhere within the genome of all isolates, raising the possibility that the Lac.1 operon, in concert with *lacG,* might still play a role in lactose metabolism. However, while the differential expression analysis showed Lac.1 to be up regulated in milk for the human isolate and NEM316 (Additional file 2), *lacG* was not upregulated. For the bovine isolate, Lac.1 showed no differential expression, which was likely due to the fact that the operon had been split into two fragments, probably due to insertion sequences that flanked one of the fragments. Similarly, Lac.1 in bovine isolate FSL S3-026 was also fragmented by insertion sequences [20]. Using BLASTn we surveyed the 202 genome sequences for the presence of Lac.1. For all isolates, we found the operon to be either contiguous, or its genes to be distributed on two or more contigs. It was contiguous in 150 isolates (human = 120, bovine = 18, tilapia = 9, bullfrog = 1, grey seal = 1, dolphin = 1), and distributed on two or more contigs in 51 isolates (human = 31, bovine = 16, tilapia = 4).

### Lactose fermentation

To further investigate the ability of different *S. agalactiae* isolates to metabolize lactose and the roles of Lac.1 and Lac.2, we performed a lactose fermentation test on a set of 124 isolates (Additional file 1). These isolates represented a subset of the 202 isolates whose genome sequences we analyzed earlier, plus the isolates analyzed here using RNAseq data and the bovine isolate FSL S3-026 (human = 74, bovine = 36, tilapia = 11, bullfrog = 1, grey seal = 1, dolphin = 1). All isolates possessing Lac.2 showed a positive result for lactose fermentation (media changed to yellow), lending further support to the role of Lac.2 in lactose metabolism. With the exception of three isolates that showed no color change, the media for all remaining isolates changed to orange, suggesting some minimal acid production and fermentation activity. Although showing positive for lactose fermentation, the genome sequence of strain FSL S3-442 lacked Lac.2. We designed a PCR test to investigate whether this was due to a sequencing omission (i.e. this particular region of the genome had not been sequenced). Primers designed within *lacF* and *lacG* amplified a ~2.5 kbp region specific to Lac.2 (*lacFEG*) confirming the operon’s presence. Although it’s possible that a similar sequencing omission could have occurred in another strain, an omission of this size is likely a rare occurrence.

Overall, our results suggest that the ability to metabolize lactose at a significant level, was acquired via the lateral transfer of the Lac.2 operon and that this was likely an important adaptation to the bovine environment. Furthermore, this mechanism permits rapid adaptation and may explain why some STs suddenly become more prevalent within the bovine environment. For example, the recent rapid increase in the prevalence of ST-1, ST-23, and ST-103 in Danish dairy herds [5]. These findings also serve to highlight how attempts to correlate adaptive traits with STs may be misleading (e.g. not all isolates of the same ST possess Lac.2).

Assuming that *S. agalactiae* acquired Lac.2 within the bovine environment, the isolation of strains from the human environment possessing Lac.2 highlights the potential for the bovine environment to serve as a reservoir for the emergence of more virulent strains with subsequent transmission to the human population. Furthermore, if LGT is the dominant adaptive process, as also proposed by Sørenson et al. [35], a dependence on MLST as the primary source of molecular epidemiological data will likely prove inadequate for accurate elucidation of these processes. For example, the proposal that the hyperinvasive neonatal ST-17 evolved via vertical inheritance from a bovine ancestor [15] was based on a phylogenetic analysis of ST data. However, more detailed genomic examination has called the accuracy of this relationship into question [12,35], and our results additionally provide no support for this relationship. Specifically, fifteen of the genome sequences surveyed here were ST-17 and none of these isolates possessed Lac.2. Therefore, our results provide no support for ST-17 being derived from a bovine adapted strain.

### Additional patterns of differential expression

There were nine genes uniquely up regulated in the bovine isolate (Table 2 and Additional file 3). Two were hypothetical proteins and in general the remainder had roles in binding and membrane transport. One of the genes involved in membrane transport was a copper-transporter ATPase *CopA*. While essential for life, copper is also highly toxic to cells in excess. The *CopA* gene product is a copper efflux ATPase involved in copper homeostasis [66,67], and disruption of the gene in *Escherichia coli* has been shown to produce sensitivity to copper [68]. Copper is present as a trace element in bovine milk [69,70] and up regulation of *CopA* may contribute to extended survival for the bovine isolate within the udder.

**Table 2:** **Uniquely up regulated genes for *S. agalactiae *** **strains NEM316 and FSL S3-586**

**Strain**	**Locus tag**	**Gene**	**Sequence description**
FSL S3-586^A^	FSLS3586_00500		Family transcriptional regulator
FSL S3-586^A^	FSLS3586_01665		Xanthine uracil permease family protein
FSL S3-586^A^	FSLS3586_04875		23S rRNA (uracil-5-)-methyltransferase
FSL S3-586^A^	FSLS3586_07316		Conjugal transfer protein
FSL S3-586^A^	FSLS3586_07998		Cyclic nucleotide-binding domain protein
FSL S3-586^A^	FSLS3586_08765	*XerS*	Site-specific tyrosine recombinase
FSL S3-586^A^	FSLS3586_09285		Hypothetical protein
FSL S3-586^A^	FSLS3586_09582		Hypothetical protein
FSL S3-586^A^	FSLS3586_10962	*CopA*	Copper-translocating P-type ATPase
FSL S3-586^B^	FSLS3586_02692	*lacG*	6-phospho-beta-galactosidase
NEM316^B^	gbs1329	*lacG*	6-phospho-beta-galactosidase
FSL S3-586^B^	FSLS3586_05359	*pyrD*	Dihydroorotate dehydrogenase 1A
NEM316^B^	gbs0553	*pyrD*	Dihydroorotate dehydrogenase 1A
FSL S3-586^B^	FSLS3586_10188	*guaC*	Guanosine 5-monophosphate oxidoreductase
NEM316^B^	gbs1154	*guaC*	Guanosine 5-monophosphate oxidoreductase
FSL S3-586^B^	FSLS3586_09702	*purF*	Amidophosphoribosyltransferase
NEM316^B^	gbs0025	*purF*	Amidophosphoribosyltransferase
FSL S3-586^B^	FSLS3586_09687		GNAT family acetyltransferase
NEM316^B^	gbs0028		GNAT family acetyltransferase
FSL S3-586^B^	FSLS3586_09712	*purC*	Phosphoribosylaminoimidazole-succinocarboxamide synthase
NEM316^B^	gbs0023	*purC*	Phosphoribosylaminoimidazole-succinocarboxamide synthase
FSL S3-586^B^	FSLS3586_02702	*lacF*	PTS lactose-specific IIA component
NEM316^B^	gbs1331	*lacF*	Lactose-specific phosphotransferase enzyme IIA component
FSL S3-586^B^	FSLS3586_02697	*lacE*	PTS lactose-specific iibc component
NEM316^B^	gbs1330	*lacE*	PTS family lactose porter iicb component
NEM316^C^	gbs0668		D-lactate dehydrogenase
NEM316^C^	gbs0789		Major facilitator superfamily protein
NEM316^C^	gbs1264		Alpha-acetolactate decarboxylase
NEM316^C^	gbs1332	*lacT*	Transcription antiterminator
NEM316^C^	gbs1508		4-alpha-glucanotransferase
NEM316^C^	gbs1619		D-3-phosphoglycerate dehydrogenase
NEM316^C^	gbs1627		CBS domain protein
NEM316^C^	gbs1630		Branched-chain amino acid ABC superfamily ATP binding cassette permease protein
NEM316^C^	gbs1631		Branched-chain amino acid ABC superfamily ATP binding cassette membrane protein
NEM316^C^	gbs1632		Branched-chain amino acid ABC amino acid-binding protein
NEM316^C^	gbs2002		Glycerol dehydrogenase

Superscript A = genes uniquely up regulated for FSL S3-586, superscript B = genes uniquely up regulated for FSL S3-586 and NEM316, superscript C = genes uniquely up regulated for NEM316.

In addition to the Lac.2 genes, there were five up regulated genes shared between the bovine isolate and NEM316 that were not up regulated for the human isolate (Table 2 and Additional file 3). These genes were all involved in metabolic processes, with four of them specifically involved in the metabolism of purine (*purC*, *purF*, *guaC*) and pyrimidine (*pyrD*). The purine and pyrimidine biosynthetic pathways have been shown to be critical for growth in human blood for other gram positive and negative bacteria [71], and our results show they may also be important for growth in bovine milk. However, Mereghetti et al. [30] found considerably greater expression for these pathways in NEM316 when grown in human blood. Specifically, ten of the 17 genes in the purine pathway and five of the six genes in the pyrimidine pathway were up regulated, suggesting a more important role during growth in blood than milk. In addition, these observations describe a distinctive metabolic contrast for NEM316 when grown in bovine milk compared to human blood. In milk, all of the genes in the Lac.2 operon were up regulated, whereas only four genes in the purine and pyrimidine biosynthetic pathways were up regulated. In contrast, growth in blood resulted in up regulation of all the genes in the purine and pyrimidine pathways and just two in Lac.2 (*lacD* and *lacE*) [30]. These observations reveal a metabolic flexibility for NEM316, where the strain can up or down regulate different metabolic pathways to various levels of expression depending on the environment.

The fifth of the up regulated genes shared between the bovine isolate and NEM316 was a GCN5-related N-acetyltransferase (GNAT). An important activity of some members of this family of genes is antibiotic (aminoglycoside) resistance [72,73]. To explore the gene’s role in *S. agalactiae* further, we performed a BLASTn search of the nr database at NCBI using the NEM316 nucleotide sequence. The top five hits were for *S. agalactiae* strains, with each hit having a similar gene annotation to NEM316. However, the next best hit was for a Zwittermicin A resistance protein *zmaR* from *Lactobacillus salivarius* (72% identity, 44% coverage), lending support to the possibility that the *S. agalactiae* GNAT gene may also be involved in antibiotic resistance. *S. agalactiae* infection is frequently treated with antibiotics [9]. Furthermore, blanket dry cow therapy (infusion of the udder with antibiotics during the dry period as a mastitis preventative measure) is now common place within the US dairy industry [74]. A commonly used antibiotic is penicillin-dihydrostreptomycin (Quartermaster; Pfizer Animal Health) [74] and dihydrostreptomycin is an aminoglycoside antibiotic. Consequently, up regulation of the putative antibiotic resistance gene might reflect an adaptation to this type of treatment where growth in milk elicits high expression of the gene for bovine adapted strains. Indeed, Brown and Scasserra [75] reported low susceptibility to streptomycin for *S. agalactiae* isolated from bovine mammary glands. An alternative explanation for up regulation of the gene is the presence of an antibiotic in the milk sample. This seems unlikely as none of the four cows providing the milk were treated with antibiotics for six months prior to milk collection. Regardless, the potential role of this gene in antibiotic resistance remains preliminary without further functional analyses.

For NEM316, there were 11 uniquely up regulated genes (Table 2 and Additional file 3). In general, these genes had roles in oxidation-reduction processes and membrane transport. One of these genes was a glycerol dehydrogenase. These enzymes are utilized in the metabolism of glycerol, which bacteria can use as a carbon source in anaerobic conditions through coupled oxidative and reductive pathways [76-78]. The triglycerides in bovine milk contain glycerol and the up regulation of glycerol dehydrogenase for NEM316 suggests that the isolate might be utilizing glycerol as a carbon source. Furthermore, glycerol-catabolizing enzymes have been shown to be important for bacterial growth [79] and NEM316 showed considerably more growth in milk than the other two isolates (Figure 1). An additional up regulated dehydrogenase was D-lactate dehydrogenase. Lactose is composed of β-D-galactose and α/β-D-glucose. After transport of lactose into the cell via the PTS, *lacG* hydrolyzes lactose-6-phosphate into both galactose-6-phosphate and glucose [80] (galactose-6-phosphate is passed to the tagatose pathway [Lac.2] as discussed above). The final product of the glycolysis of glucose is pyruvate, and when oxygen is absent or in short supply, D-lactate dehydrogenase converts pyruvate to lactate [81]. Consequently, up regulation of D-lactate dehydrogenase suggests active metabolism of the glucose component of lactose for NEM316. Glucose metabolism is also indicated by the up regulation of α-acetolactate decarboxylase, which is involved in the anabolism of acetoin from pyruvate [82]. This gene activity might again contribute to strain NEM316’s stronger growth in milk.

### Nisin and insertion sequences

*Streptococcus uberis* possesses an 11-gene operon for the production of the lantibiotic nisin [83]. The operon is part of the species' dispensable genome, [83] and Pryor et al. [84] showed that nisin producer strains dominated non-producer strains during intramammary infection, suggesting a competitive advantage. *S. agalactiae* (stain FSL S3-026) also possesses the operon [20]. However, the operon is disrupted by an insertion sequence (IS) and a deferred antagonism test showed the strain to not produce nisin. The bovine *S. agalactiae* isolate studied here (strain FSL S3-586) also possessed the operon; but again the operon was disrupted by insertion sequences (fragmented into two). However, all 11 operon genes showed some expression in milk, whereas only five showed expression in broth. However, this expression was not significantly different between the two environments and a previous deferred antagonism test showed the strain to not produce nisin [20]. Similar to strain FSL S3-026, strain FSL S3-586 also possessed numerous insertion sequences (122) throughout its genome, lending further support to the importance of IS activity in the evolution of *S. agalactiae*. A total of 106 (86.9%) of the 122 IS showed no gene expression in either milk or broth, while 12 IS were expressed in both environments, although not differentially, suggesting some IS activity in both environments. Four IS showed negligible expression in milk only. The human isolate contained considerably fewer IS (42). However, there was a similar number of IS showing expression in both environments (11). The lower number of IS for human isolates may be typical, as the average number of IS for the human *S. agalactiae* isolates 2603 V/R, H36B, 18RS21, A909, 515, CJB111, and COH1 was 23 [20]. NEM316 is somewhat distinctive in that it possesses a comparatively very low number of IS (5), with three showing expression in both environments. The high number of IS for the bovine isolates might reflect a degree of specialization to this environment. For example, several studies have suggested that proliferation of insertion sequences is an evolutionary signature that accompanies the transition to a more specialized life style [85-88]. Specifically, in any population, the transposition of IS into genes occurs at a particular rate, and in large populations, if a transposition is lethal or results in a selective disadvantage, the bacterial will be removed from the population via purifying selection. However, the reduction in population size that typically accompanies specialization increases the effect of genetic drift, which is now able to fix more of the deleterious transpositions into the population. The very low number of IS for NEM316 might reflect a larger population size associated with a more generalist ability (ST-23 has the widest reported host distribution).

## Conclusion

Our study detected numerous genetic factors likely important in *S. agalactiae*’s adaptation to the bovine environment. In particular, the acquisition of Lac.2 and the ability to efficiently metabolize lactose appears to have been a major adaptation. We provide convincing evidence supporting LGT as the mechanism responsible for this adaptation, and rather than being a single evolutionary event, it appears to have occurred multiple times, with all *Streptococcus* species known to be major causes of mastitis identified as possible donors. This process has resulted in genetically divergent types of Lac.2 within *S. agalatiae* warranting further investigation into the possible affects this has on survivability and the propensity to cause mastitis. Other factors such as up regulation of genes involved in copper homeostasis, and metabolism of purine, pyrimidine, glycerol and glucose were also specific to bovine adapted strains. Although somewhat speculative without further functional studies, the up regulation of a GNAT gene during growth in milk that may impart antibiotic resistance, was of particular interest, as it hinted at an adaptation to the use of antibiotics within this environment. Other mastitis causing pathogens are less responsive to antibiotics [10] and the evidence provided here and elsewhere, for LGT between *S. agalactiae* and these species, highlights the potential for further development and spread of antibiotic resistance.

### Availability of supporting data

The Illumina derived short read files are available at the NCBI Sequence Read Archive (SRA) under the study accession number SRP026339.

## Competing interests

The authors declare that they have no competing interests.

## Authors’ contributions

VPR and MJS conceived the project; VPR conducted data analysis and wrote the manuscript; MJS provided the conceptual framework, helped write the manuscript, and secured funding; MJS and PDPB provided experimental design; SCC designed and implemented the RNAseq analysis pipeline; PDPB conducted laboratory work associated with bacteria culturing, lactose fermentation, and transcriptome sequencing; AAG provided milk samples and helped design laboratory protocols. All authors read and approved the final manuscript.

## Supplementary Material

Additional file 1 Strain information. Click here for file

Additional file 2 Results of differential expression analysis and GO terms. Click here for file

Additional file 3 Uniquely up regulated genes for NEM316 and FSL S3-586. Click here for file
